# An Obscure Case of Peri-Uterine Cellulitis

**Published:** 1886-10

**Authors:** W. C. Fisher

**Affiliations:** Galveston


DANIEL'S
TEXAS MEDICAL JOURNAL.

PUBLISHED MONTHLY. F. E. DANIEL, M. D, Editor and Pub'r. SUBSCRIPTION $2.00 A YEAR.

Vol. 2 AUSTIN, OCTOBER, 1886. No. 4.

### ORIGINAL ARTICLES.

CONTRIBUTED EXCLUSIVELY TO THIS JOURNAL.

*The Articles in this Department are accepted and published with the understanding that we are not responsible for, nor do we indorse the views and opinions of the writers, by so doing.*

### AN OBSCURE CASE OF PERI-UTERINE CELLULITIS.

### Mistaken for Pregnancy of Three or Four Months' Duration; with Remarks on Diagnosis and Treatment.

*By W. C. Fisher, M. D., Galveston.*

[For Daniel's Texas Medical Journal.]

B. W., age 22 years, nativity Wisconsin, called at my office July 2, 1886, about 3 p. m., with the following history: Mother of two children. The first was born when she was seventeen years old; the second, two years later. Both labors were normal. Has had two abortions induced, in what manner I am unable to state. There was only two months interval between the abortions. She was anæmic, and presented the appearance ot one who had suffered, both mentally and physically. Her statement was, that for the past three weeks she had been flooding a good deal; had pains resembling labor pains, and her womb was open. On inquiry, I ascertained that she was of the opinion that she was pregnant, and had introduced a pen-holder into the uterus and worked it around with considerable force, until

(she said that) there was a faint feeling and some nausea. I ordered her a prescription containing ergot, viburnum, bromide of potassium and morphia, a combination which I have found very useful in cases presenting similar symptoms. In addition to this I prescribed rest in the recumbent posture. I was sent for the next evening, and found her suffering with pains similar to the *wagging pains* of the first stage of labor. On digital examination, I found the vagina and adnexa rather warmer than normal, but not unduly sensitive to touch. The uterus was slightly prolapsed, and the external os was sufficiently patulous to admit the index finger up to the first joint; and in Douglass' *cul-de-sac* there was a hard mass the size of an orange, which, on pressure, produced scarcely any pain. From this, I came to the conclusion that there was a fœtus in the uterus which was very anxious to come out. Whether it had been destroyed by the rough treatment it had had, or was still alive, I was unable to determine, she not being far enough advanced ia pregnancy to hear the fœtal heart. I ordered powdered opium, in grain doses, every two hours, until the pain was relieved, and impressed upon her the importance of keeping perfectly quiet. I called the next morning and found her free of fever, and was told that she spent a comfortable night, and that she was feeling much better in every respect. I left instructions for her to keep quiet and take no medicine unless the pain returned. Late that evening I was sent for, and found her suffering so much pain that I was forced to give opium freely. Up to this time there had been no fever. Her symptoms and physical signs not changing for the better, I felt assured that the fœtus was dead, and, if not, her condition was such that the induction of labor was justifiable. In this, my friend, Dr. Sampson, agreed with me, and gave most valuable assistance throughout the case. I first tried to introduce a Simpson's sound, but after several ineffectual attempts I desisted. I then, after patient trial, introduced a Sims' sound, which bent at the half of a circle posteriorly—(the depth was about three inches). Dr. Sampson then drew the uterus well down with a tenaculum, and introduced a pair of long uterine dressing forceps, and gave the os a good stretching. The patient was left some morphia and ordered ergot pretty freely, and hot vaginal irrigations. That evening, late, I found her with some fever, and right well under the influence of the anodyne. The next evening the fever had disappeared, and on vaginal examination, the parts were normal to touch, and if anything, the external os less patulous than it was prior to the stretching.

Her condition remaining about the same for four days, and nothing escaping from the uterus in the shape of a fœtus, and she being very anxious to have the womb relieved of its contents, I had her thoroughly anæsthetized, and then introduced a uterine dilator. After this I carried quite a large piece of anti-septic cotton well into the uterus, and let it remain. When the patient recovered from the effects of the chloroform, I ordered the free use of ergot, in the hope that it would increase the action of the uterus, and expel the mass through the dilated os. I also ordered frequent hot antiseptic injections. In a few hours after performing this opertion, the pulse ran up to about 120, and the temperature to 103 1-2; but, under the influence of large doses of quinine and opium, I soon brought it down two degrees. In abou thirty-six hours, there being quite a fetid odor in the womb, which evidently originated in the uterus, I removed the pledget of cotton, and, to my surprise, a large quantity of fetid pus escaped. There must have been from one-half pint to a pint. At first I thought that it was due to decomposition of the fœtus, but when I called the next day, I found her free of fever, and on examination, the hard mass in Douglass' *cul-de-sac* had diminished a great deal; then, for the first time, I recognized my case as one of *periuterine cellulitis*, with the opening of the abscess into the uterus, instead of a case of pregnancy. Under the influence of a gentle tonic and hot injections, she made a rapid and uninterrupted recovery.

She then told me that she felt pretty sure that she passed the fœtus before coming to my office, but that, not being certain, she wished to deceive me, in the hope that I could remove it if it still remained. The cause of the cellulitis was undoubtedly the means which she used to induce the abortion.

REMARKS.

Dr. Thomas, in his most excellent work, says: "The diseases with which cellulitis may be confounded, are: Fibrous tumors, hematocele, pelvic peritonitis and *early pregnancy*." He gives the following symptoms: Chill, increased thermometric range, pain, fever, dysuria, metrorrhagia. He further says: "But he who awaits these symptoms for diagnosis, will be led into many errors of omission, for subacute cases very generally, and acute cases sometimes, fully develop themselves without them." He says: "All cases may be brought under three heads as to severity of symptoms: 1st. Cases accompanied by chill, fever, pain, and the ordinary signs of inflammation. 2d. Those accompanied by pain without chill or fever. 3d. Those marked by scarcely any symptoms, except extreme feebleness and some sense of pulsation, and weight about the pelvis, with hectic fever towards evening."

In another place, in regard to the escape of the purulent matter, he says: "1st. Through the abdominal walls or saphenous openings. 2d. Through the pelvic viscera, bladder, rectum, vagina, urethra, or *uterus* (italics mine). 3d. Through floor of the pelvis, near the anus. 4th. Through the pelvic foramina, obturator, or sacro-ischiatic. 5th. Through pelvic roof into the peritoneal cavity."

He also says, "The most frequent channels of evacuation are the vagina and rectum, in the non-puerperal form, and probably the abdominal walls in the puerperal, or, at least, the results of Dr. McClintock's carefully noted cases would lead us to believe so. In thirty-seven puerperal cases treated by him, which ended in suppuration, twenty abscesses discharged in the iliac regions, two above the pubes, one in the inguinal region, and one beside the anus. Of the remaining thirteen, six were discharged per vaginam, five per anum, and two burst into the bladder."

My object in reporting this case, is because I think my error in diagnosis was perfectly justifiable, and because it presents several interesting features. My reasons for thinking the error in diagnosis justifiable, are the following:

1. The history as given by the patient, and the large mass in the *cul-de-sac* of Douglass, together with the patulous condition of the os, the peculiar pains which were similar to labor pains, and the hemorrhage. These symptoms, surely, are very suggestive of an impending or threatened miscarriage.

2. The absence of a marked chill, which generally ushers in a case of cellulitis.

3. No rise of temperature, save that which followed operative measures.

4. There was no dysuria, which is generally quite well marked in cellulitis.

5. There was metrorrhagia, which is generally a marked symptom of cellulitis; but, on the other hand, is it not the rule, with scarcely an exception, that we have hemorrhage in a case of abortion, prior to the expulsion of the fœtus?

6. On digital examination, there was scarcely any undue heat in the vagina and adjacent organs, and in cellulitis there is generally quite an elevation in the local temperature. Neither was there undue tenderness on pressure, either in the vagina or against the mass, while in cellulitis, tenderness is a very prominent symptom.

A most interesting feature in this case is the discharge of the pus through the uterus. This is, indeed, a very rare occurrence, as you will see by reference to the quotations from Dr. Thomas, which I have given above.

Another very interesting point in the case is, that my patient ever survived the active treatment which she was subjected to while suffering with cellulitis.

If this case will be of interest to the readers of the JOURNAL, I will feel fully repaid for the trouble I have had in preparing it.

